# Mechanism of *Astragalus membranaceus* in the treatment of laryngeal cancer based on gene co-expression network and molecular docking

**DOI:** 10.1038/s41598-020-68093-0

**Published:** 2020-07-07

**Authors:** Kai Feng Dong, Meng Qi Huo, Heng Ya Sun, Tian Ke Li, Dan Li

**Affiliations:** 1 Department of Otolaryngology, The First Hospital of Hebei Medical University, Shijiazhuang, 050000 China; 2 Department of Otolaryngology, The Third Hospital of Shijiazhuang, Shijiazhuang, 050011 China; 3 Department of Stomatology, The Fourth Hospital of Hebei Medical University, Shijiazhuang, 050011 China; 4 School of Chinese Materia Medica, Beijing University of Chinese Medicine, Beijing, 102488 China

**Keywords:** Oral cancer, Computational biology and bioinformatics

## Abstract

*Astragalus membranaceus* (HUANG QI, HQ) is a kind of traditional Chinese medicine. Researchers have widely concerned its antitumor effect. At present, there is still a lack of research on the treatment of laryngeal cancer with HQ. In this study, we integrated data from the weighted gene co-expression network of laryngeal cancer samples and the components and targets of HQ. A new method for dividing PPI network modules is proposed. Important targets of HQ treatment for laryngeal cancer were obtained through the screening of critical modules. These nodes performed differential expression analysis and survival analysis through external data sets. GSEA enrichment analysis reveals pathways for important targets participation. Finally, molecular docking screened active ingredients in HQ that could interact with important targets. Combined with the laryngeal cancer gene co expression network and HQ PPI network, we obtained the critical module related to laryngeal cancer. Among them, MMP1, MMP3, and MMP10 were chosen as important targets. External data sets demonstrate that their expression in tumor samples is significantly higher than in normal samples. The survival time of patients with high expression group was significantly shortened, which is a negative factor for prognosis. GSEA enrichment analysis found that they are mainly involved in tumor-related pathways such as ECM receptor interaction and Small cell lung cancer. The docking results show that the components that can well bind to important targets of HQ are quercetin, rutin, and Chlorogenic acid, which may be the primary mechanism of the anti-cancer effect of HQ. These findings provide a preliminary research basis for Chinese medicine treatment of laryngeal cancer and offer ideas to related drug design.

## Introduction

Laryngeal cancer is one of the common malignant tumors of the head and neck, most of which are squamous cell carcinoma^1^. It ranks second in the incidence of head and neck cancer (HNSC). The affected population is mainly middle-aged and older men over 40 years old, with a slight trend of youth^2^. The occurrence and development of laryngeal cancer are related to many factors, including smoking, drinking, HPV infection, radiation, low intake of vegetables and fruits, insufficient trace elements, and disorders of sex hormone metabolism^3–5^. In the early stage of laryngeal cancer, laser treatment has less damage and less postoperative complications^6^. For most patients with laryngeal cancer, open surgery is still the main treatment. Radiotherapy and chemotherapy were used as auxiliary means^7^. Targeted treatment of tumor can increase drug sensitivity, improve curative effect and reduce side effects^8^. However, not all patients can benefit from it^9^. Clearing tumors, preserving function, controlling recurrence, and improving quality of life are the goals of treating laryngeal cancer^5^.

*Astragalus membranaceus* (HUANG QI, HQ) is one of the most commonly used Chinese medicine in clinical practice, which is the dried rhizome of *Astragalus membranaceus*. It has been reported that HQ can enhance immunity and protect cardiovascular system^10^. In recent years, the antitumor effect of HQ has attracted widespread attention from researchers^11^. On the one hand, it can enhance cellular and humoral immune functions, improve the killing and inhibiting effects on tumor cells, and promote tumor cell apoptosis^12^. On the other hand, it can also reduce the damage to the immune function of radiotherapy and chemotherapy, and play a role in the adjuvant treatment of tumors^13^. However, there are few related studies on HQ in treating laryngeal cancer.

In recent years, researchers have studied the pathogenesis of laryngeal cancer at the molecular level to find more effective treatments^14^. Network pharmacology is a new discipline based on systems biology theory, selecting specific signal nodes for multi-target drug design^15^. It emphasizes multi-channel regulation of signal pathways, improves the therapeutic effect of drugs, and reduces toxic and side effects^16^. This method can improve the success rate of clinical trials of new drugs and save drug development costs^17^. The protein–protein interaction (PPI) network is a common technique to analyze the mechanism of traditional Chinese medicine and disease from the molecular level^18^. The functional modules in the protein-interaction network are composed of interacting proteins, which together participate in specific biological processes^19^. Therefore, analyzing the functional modules in the network is of great significance for studying the mechanism of HQ treatment of laryngeal cancer. In previous studies, people often use the MCODE algorithm, FAG-EC algorithm, etc. to divide modules in PPI networks. Most of these algorithms divide modules based on the heterogeneity of scale-free networks^20,21^. They can obtain modules with tighter connectivity on the network topology. However, we prefer to get modules that are more closely connected to biological functions^22^. In this study, we designed a method for obtaining modules that integrate disease gene expression data and clinical information. Based on the gene expression data of 109 laryngeal cancer samples, we constructed a weighted gene co-expression network of laryngeal cancer. The modules are divided according to the correlation between genes, and then they are mapped in the PPI network of related targets of HQ. In this way, modules with a high correlation in biological functions can be received, and redundant PPI data in the protein interaction network can be removed. This method makes the selection process of critical nodes more concise and accurate^23^.

In this study, we propose a new method for obtaining PPI network modules. Based on the gene expression information in the GSE27020 dataset, a weighted gene co-expression network was constructed. The co-expressed gene modules were mapped into the PPI network of HQ, and critical genes and corresponding targets were obtained by screening. These genes were confirmed to be differentially expressed in the tumor group and the normal group, and the survival time of patients with high expression group was significantly shortened. Based on the docking, the active ingredients in HQ were obtained. These findings may provide a new perspective for the treatment of laryngeal cancer with HQ.

## Results

### Acquisition of laryngeal cancer samples

The GSE27020 data set contains a total of 109 laryngeal cancer samples. After removing the genes with too many missing values from samples, an expression matrix containing 13,512 genes was generated. The 3,378 genes with the top 25% of the variance (that is, the genes that change significantly in each sample) are selected for subsequent operations. Clinical phenotypic data included survival time, recurrence, age, and tumor stage. As shown in Fig. S1A. According to the distance of sample clustering, the outliers GSM665579, GSM665597, and GSM665566 were eliminated. Finally, we selected 106 samples to construct a weighted gene co-expression network.

### Identification of critical modules

**Figure 1:**
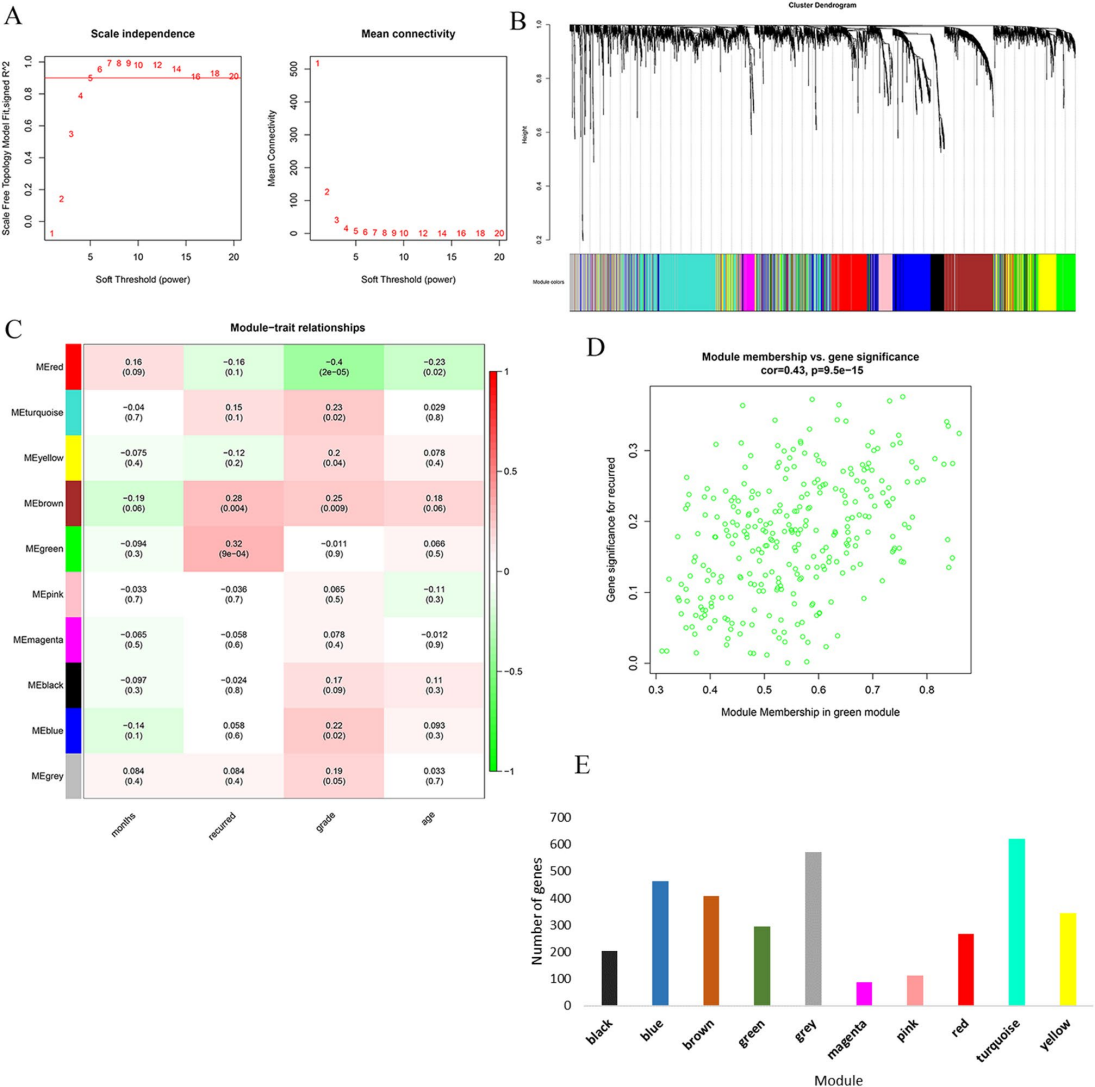
Drawing weighted gene co expression network of laryngeal cancer based on WGCNA package of R 3.5.1 software (https://www.r-project.org/) (**A**) Analysis of the scale-free fit index for various soft-thresholding powers (β), 5 was the most fit power value. (**B**) The cluster dendrogram of genes in GSE27020. Each branch in the figure represents one gene, and every color below represents one co-expression module. (**C**) Heat map of the relationship between the characteristic genes of modules and the incidence of laryngeal cancer based on R 3.5.1 software. Correlation coefficient along with P value in parenthesis underneath. Color-coded according to correlation coefficient (legend at right). (**D**) Scatter plot of gene significance and module membership in the green module. (**E**) The number of genes in each module.

We use the WGCNA package in R software to build a weighted co-expression network^24^. When R^2^ > 0.9, the network meets the scale-free condition. The calculation result is shown in Fig. 1A, where SFT.R.sq is R^2^. When the power value is set to 5, the connectivity between genes in the network satisfies the scale-free network distribution. We use a dynamic cutting algorithm to obtain ten modules, of which the gray module is a set of genes that cannot be aggregated into other modules (Fig. 1B). Then, the correlation between each module and the phenotype (survival time, recurrence, age, and grade) was calculated (Fig. 1C). A heat map showed a significant positive correlation between the green module and recurrence (cor = 0.32, P < 0.001) (Fig. 1D). The number of genes in each module is shown in Fig. 1E. The GS and MM scatter plots of the green module also illustrate the significant correlation between the green module and laryngeal cancer. Therefore, the green module was selected as the critical module.

### Enrichment analysis of the critical module

**Figure 2:**
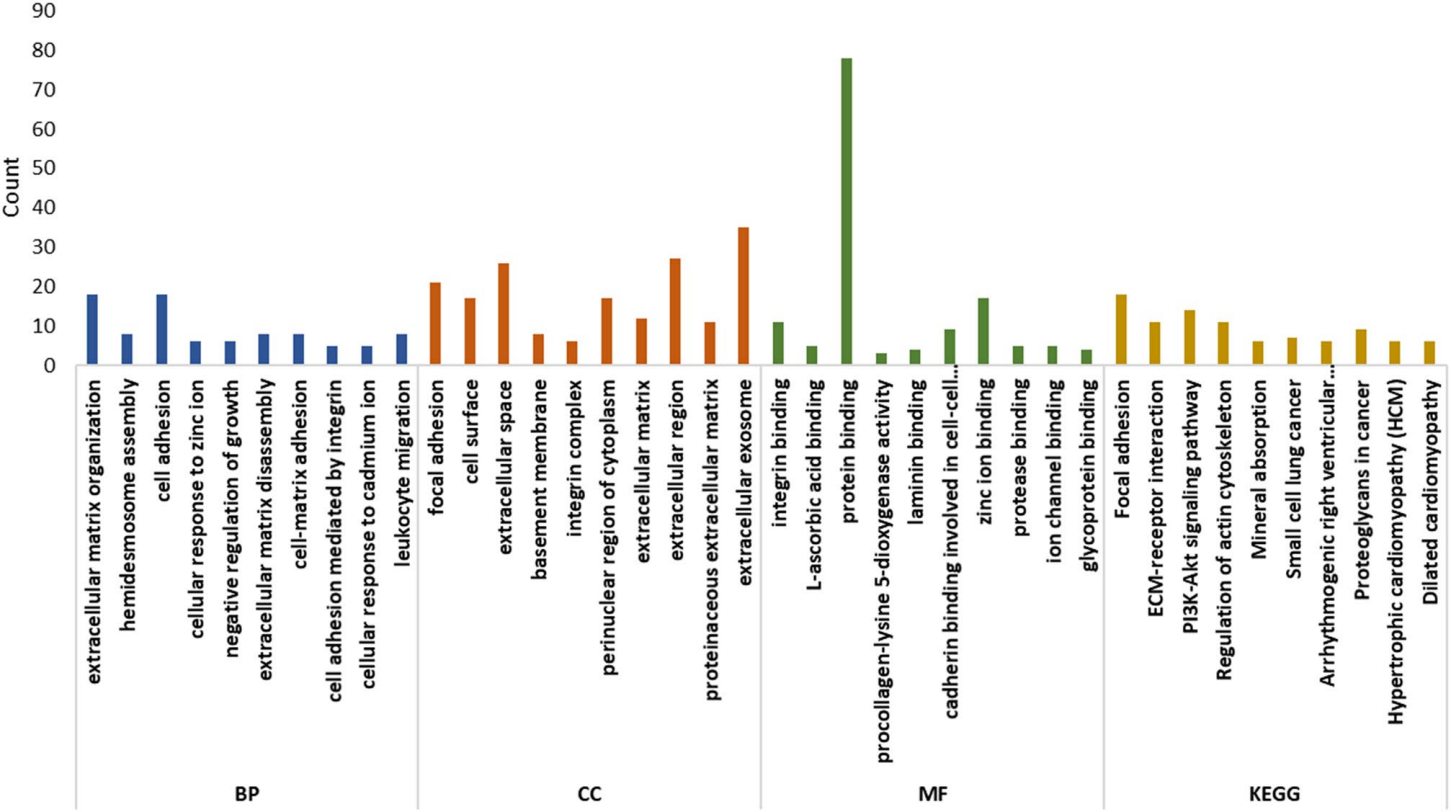
GO and KEGG enrichment analysis of green module based on DAVID 6.8 database (https://david.ncifcrf.gov/). X-axis refers to the ID of the enriched terms, and Y-axis refers to the number of the enriched genes. Brown bars show the results of the biological process classification. Blue bars show the results of the cellular component classification. Yellow bars show the results of the molecular function classification. Green bars show the results of the KEGG pathway classification.

We used GO enrichment analysis and KEGG pathway analysis to find the biological function of genes in the green module (Fig. 2)^25,26^. In the biological process classification, genes in the module are significantly enriched in extracellular matrix organization, hemidesmosome assembly, cell adhesion, cellular response to zinc ion, negative regulation of growth, extracellular matrix disassembly, cell–matrix adhesion, cell adhesion mediated by integrin, cellular response to cadmium ion, leukocyte migration. In the cellular component classification, the genes in modules are significantly enriched in focal adhesion, cell surface, extracellular space, basement membrane, integrin complex, perinuclear region of cytoplasm, extracellular matrix, extracellular region, proteinaceous extracellular matrix, extracellular exosome. In the molecular function classification, the genes in modules are significantly enriched in integrin binding, L-ascorbic acid binding, protein binding, procollagen-lysine 5-dioxygenase activity, laminin binding, cadherin binding involved in cell–cell adhesion, zinc ion binding, protease binding, ion channel binding, glycoprotein binding. In the KEGG pathway classification, the genes in modules are significantly enriched in focal adhesion, ECM-receptor interaction, PI3K-Akt signaling pathway, regulation of actin cytoskeleton, mineral absorption, small cell lung cancer, arrhythmogenic right ventricular cardiomyopathy (ARVC), proteoglycans in cancer, hypertrophic cardiomyopathy (HCM), dilated cardiomyopathy. The detailed results are shown in Table S1.

### Identification of important targets

**Figure 3:**
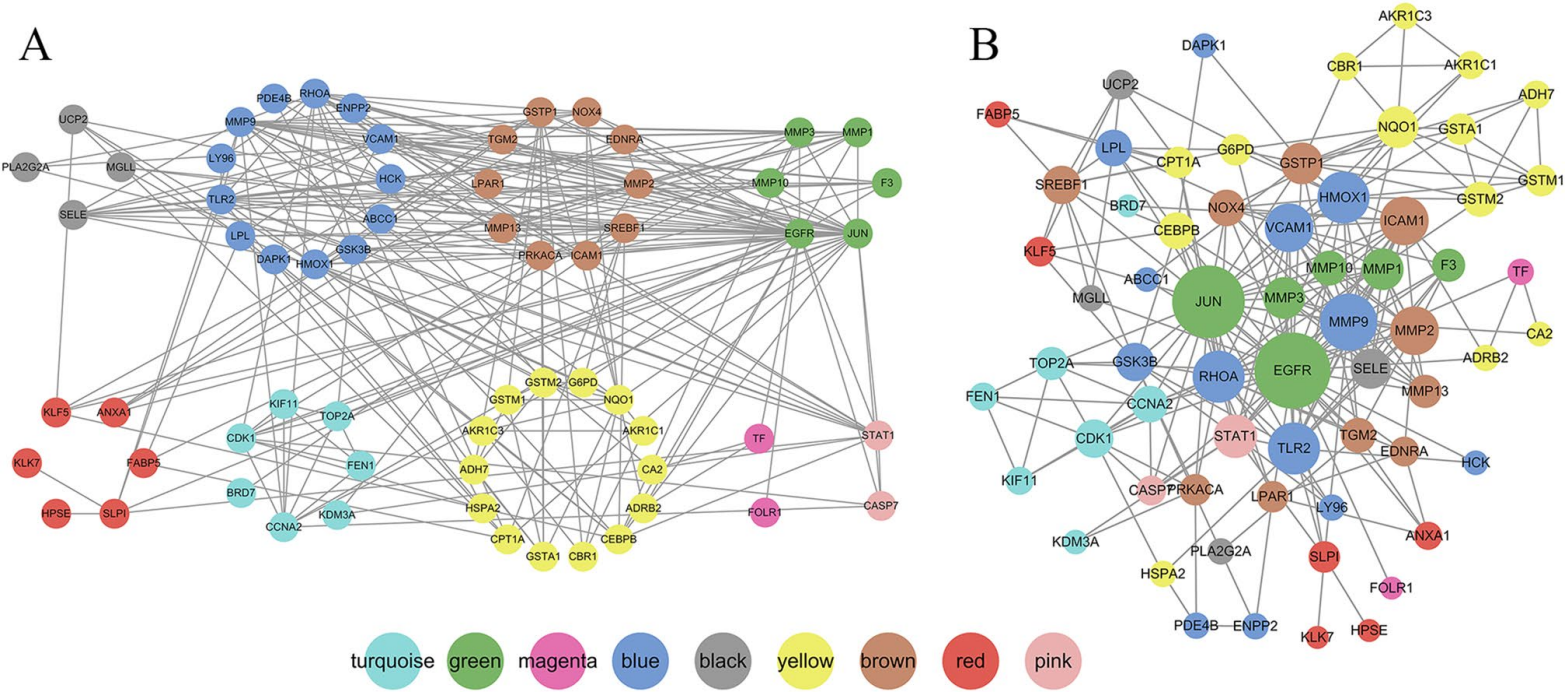
Constructing network based on Cytoscape 3.7.0. (https://cytoscape.org/) (**A**) Module diagram of laryngeal cancer weighted gene co-expression network mapped to HQ PPI network. The different colors in the figure represent the corresponding modules. The black line represents the PPI relationship between the nodes. (**B**) A larger node indicates a greater degree of the node.

A total of 77 components of HQ and 953 corresponding targets were obtained from the TCMSP database. The target's PPIs were introduced into Cytoscape to obtain the PPI network of HQ. The network contains 471 nodes and 2,983 edges, as shown in Fig. S2. We take HQ-PPI network as the background. Gene interactions in the weighted gene co expression network of laryngeal cancer was mapped to the HQ-PPI network. Common nodes and interactions between gene modules and PPI network are preserved. Finally, we got laryngeal cancer-HQ-PPI network. The network consists of 64 nodes and 249 edges, which are composed of 9 modules (with the gray modules removed), as shown in Fig. 3A. Therefore, the corresponding green module after the mapping is considered to be a critical module for HQ treatment of laryngeal cancer. Nodes in the green module are considered candidate important targets. By calculating the topology parameters of the network nodes, the degree value of each node is obtained. As shown in Fig. 3B, compared with the nodes of other modules, the nodes of the green module have a higher degree value and are in the core position of the network.

### Differential expression analysis, survival analysis, and GSEA analysis of important targets

**Figure 4:**
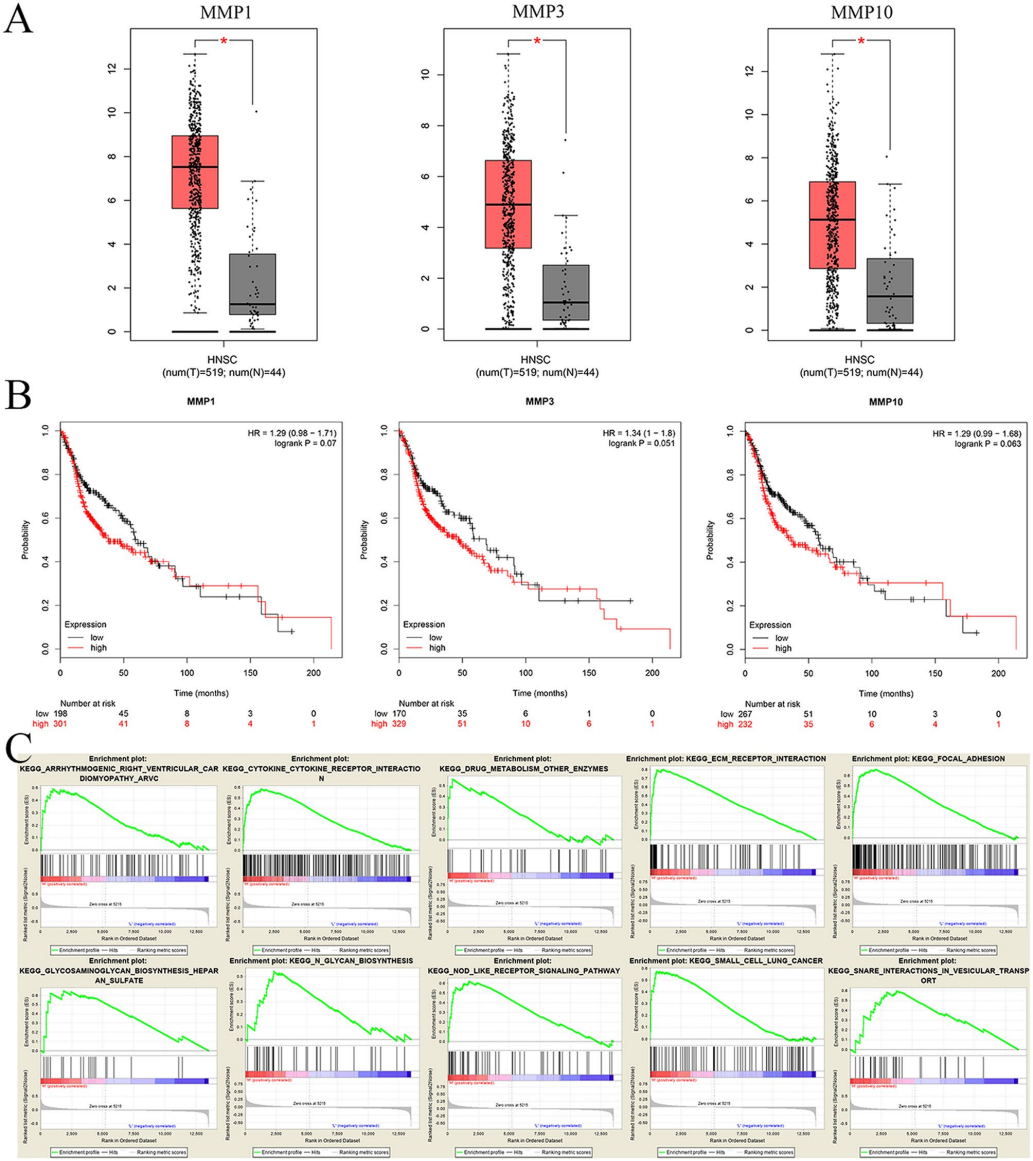
(**A**) Differential expression analysis of important targets based on TCGA database and GTEx database. (**B**) Survival analysis of important targets. Notes: three important targets in green module with significant results of survival analysis (P < 0.1 was regarded as significant). They were MMP1, MMP3, MMP10 respectively. (**C**) Enrichment analysis of important targets in high expression group based on GSEA 3.0 software (https://software.broadinstitute.org/gsea/downloads.jsp).

Based on the sample information of TCGA and GTEx projects in the GEPIA database, it was found that some candidate important targets had differential expression between normal and laryngeal cancer. The Wilcoxon rank test was performed on the normal group and the laryngeal cancer group (Fig. 4A). Among them, the expressions of MMP1, MMP3, and MMP10 in tumor tissues were higher than those in normal tissues (p < 0.05). After that, we used the Kaplan Meier-plotter website for survival analysis. The results are shown in Fig. 4B, in which MMP1, MMP3, and MMP10 were significantly correlated with patient prognosis (P < 0.1). As their expression levels increase, the overall survival time decreases significantly. The above results suggest that MMP1, MMP3, and MMP10 are poor prognostic factors, and their high expression is significantly associated with shortened patient survival. Therefore, they are considered to be important targets closely related to laryngeal cancer, which may be the primary mechanism of HQ treatment of laryngeal cancer. To understand the pathways involved in these important targets (MMP1, MMP3, MMP10), a GSEA analysis was performed using the data set GSE27020. There were ten pathways enriched in the high expression group(Fig. 4C), which are the snare interactions in vesicular transport, focal adhesion, ECM receptor interaction, glycosaminoglycan biosynthesis heparan sulfate, cytokine cytokine receptor interaction, N glycan biosynthesis, small cell lung cancer, arrhythmogenic right ventricular cardiomyopathy (ARVC), Nod-like receptor signaling pathway, drug metabolism other enzymes.

### Molecular docking

**Figure 5:**
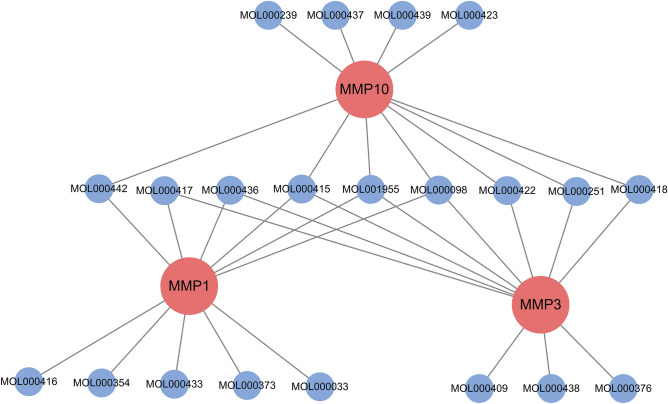
Constructing compound-target interaction network diagram based on Cytoscape 3.7.0. (https://cytoscape.org/). The blue nodes are top ten compounds with free binding energy for each target. The red nodes are important targets. The black lines indicate the relationship between compounds and targets.

A total of 77 HQ components were obtained through the TCMSP database, and the component information is shown in Table S2. The crystal structure with the PDB code of 3AYK was selected as the docking model of MMP1, and the central axis was adjusted to 3.816, -4.348, -0.55 for MMP1. The crystal structure with the PDB code of 4G9L was selected as the docking model of MMP3, and the central axis was adjusted to 21.36, 68.883, 106.078 for MMP3. The crystal structure with the PDB code of 1Q3A was selected as the docking model of MMP3, and the central axis was adjusted to 25.304, − 10.345, 24.278 for MMP10. All components are sequentially docked with MMP1, MMP3, and MMP10 to obtain the corresponding binding free energy. The calculation results of compounds and targets in the top ten (including all the 10th in parallel) are shown in Table 1 and Fig. 5. The docking results of other compounds are shown in Table S3.

**Table 1:** Top ten compounds with free binding energy for each target.

MMP1	Affinity (kcal/mol)	MMP3	Affinity (kcal/mol)	MMP10	Affinity (kcal/mol)
MOL000415	− 9.6	MOL000098	− 9.6	MOL000437	− 8.9
MOL001955	− 9	MOL000438	− 9.6	MOL000415	− 8.8
MOL000098	− 8.8	MOL000436	− 9.4	MOL000418	− 8.8
MOL000417	− 8.8	MOL000409	− 9.4	MOL000098	− 8.7
MOL000442	− 8.8	MOL000415	− 9.3	MOL000442	− 8.4
MOL000433	− 8.8	MOL000417	− 9.3	MOL001955	− 8.3
MOL000373	− 8.8	MOL000418	− 9	MOL000422	− 8.3
MOL000436	− 8.7	MOL001955	− 9	MOL000251	− 8.3
MOL000354	− 8.7	MOL000422	− 9	MOL000423	− 8.3
MOL000416	− 8.7	MOL000251	− 9	MOL000239	− 8.3
MOL000033	− 8.7	MOL000376	− 9	MOL000439	− 8.3

As shown in the figure, among the top 10 ingredients, the best combination with MMP10 is isomucronulatol-7,2′-di-O-glucosiole, Jaranol, rhamnocitrin-3-O-glucoside, Hirsutrin, the best combination with MMP3 is (3R)-3-(2-hydroxy-3,4-dimethoxyphenyl)chroman-7-ol,7,2′-dihydroxy-3′,4′-dimethoxyisoflavone-7-O-β-d-glucoside,AstragalosideIV, and the best combination with MMP1 is (2S)-2-[[4-[(2-Amino-4-oxo-1H-pteridin-6-yl)methylamino]benzoyl]amino]pentanedioic acid, (3S,8S,9S,10R,13R,14S,17R)-10,13-dimethyl-17-[(2R,5S)-5-propan-2-yloctan-2-yl]-2,3,4,7,8,9,11,12,14,15,16,17-dodecahydro-1H-cyclopenta[a]phenanthren-3-ol, Lariciresinol, Isorhamnetin, (2S)-4-methoxy-7-methyl-2-[1-methyl-1-[(2S,3R,4S,5S,6R)-3,4,5-trihydroxy-6-methylol-tetrahydropyran-2-yl]oxy-ethyl]-2,3-dihydrofuro[3,2-g]chromen-5-one. Some ingredients have stronger binding effects with three targets, such as quercetin, rutin, Chlorogenic acid.

## Discussion

In this study, we obtained ten modules based on 109 laryngeal cancer expression data in the GSE27020 dataset. The green module is the critical module we found. The results of enrichment of GO and KEGG also showed the mechanism of laryngeal cancer. Among them, cell adhesion mediated by integrin, leukocyte migration, ECM-receptor interaction, PI3K-Akt signaling pathway etc. have been proved to be closely related to the occurrence of cancer. Integrin mediates the interaction between cell and cell, cell and extracellular matrix, and participates in the information transmission inside and outside cell^27^. It plays an important role in the proliferation, differentiation, adhesion, migration and apoptosis of cancer cells. Some studies have shown that integrin is the key to cancer cell proliferation. When cancer cells float, integrin will change its function from adhesion to communication^28^. The dysfunction of ECM-receptor interaction may lead to the abnormal activation of signal transduction pathways, including Ras/ Raf/MAPK, Raf/JNK, Rho/RAC/Pak and PI3K/Akt/mTOR. So as to create a tumor microenvironment that can promote tumor survival, angiogenesis and invasion^29^. PI3K-Akt signaling pathway is the most common dysregulated pathway in many tumor cells. When it is activated abnormally, many tumor suppressor genes, including PIK3CA, PIK3R1, PTEN, Akt, TSC1, TSC2, LKB1, mTOR, will be affected^30^. The results of the enrichment analysis may be the main mechanism of laryngeal cancer.

The green module was mapped on the HQ PPI network, and finally, the potential critical targets MMP1, MMP3, and MMP10 for the treatment of laryngeal cancer were obtained. Gene expression data from the external data set showed that the expression of these three genes in the tumor group was significantly higher than in the normal group. Survival analysis showed that high expressions of MMP1, MMP3, and MMP10 would lead to a reduction in patient survival. They are detrimental to prognosis. GSEA analysis showed that the MMP1, MMP3, and MMP10 high-expression groups participated in tumor-related ECM receptor interaction, small cell lung cancer, and other pathways. MMP1, MMP3, and MMP10 belong to the matrix metalloproteinases (MMPs) family^31^. The MMPs family contains multiple types of proteases. They can break down most of the extracellular matrix components and some proteins, producing growth factors essential for tumorigenesis and progression. Therefore, the MMP family has become a biomarker for some tumors^32^. MMP1 expression increases in malignant tumor cells, which is helpful for tumor cell invasion and metastasis^33^. Studies have shown that the genetic characteristics of MMP1 may become an independent prognostic factor for laryngeal cancer. And the expression of MMP1 in most of the laryngeal cancer tissues with lymphatic metastasis was higher than that of the laryngeal cancer tissues without lymphatic metastasis. MMP1 also increased with the increase of infiltration^34^. MMP3 is one of the most active members in MMPs, and it is expressed in tumor cells such as gastrointestinal tumors and liver tumors^35^. Gobin Emily's research found that MMP3 and MMP10 were significantly up-regulated in at least ten cancer types^36^. Alaseem Ali's research shows that the MMPs family plays a vital role in the invasion and metastasis of malignant tumors and the formation of new blood vessels. Inhibiting the expression and activity of MMPs can provide new ideas for the comprehensive treatment of tumors^37^. In addition, The secretion of MMP1 can stimulate the secretion of other MMPs (e.g., MMP3, MMP10), which may be the reason why they together become a critical gene of laryngeal cancer^38^.

The docking results show that quercetin, rutin, and Chlorogenic acid can be combined with MMP1, MMP3, and MMP10. Previous studies have shown that quercetin induces apoptosis in human Hep2 cells by inhibiting Akt / PKB phosphorylation. The effect of 40 μM quercetin on the induction of apoptosis in HeP2 cells increased from 18.7 to 42.2%^39^. Rutin can inhibit the activity of NF-kappa B and P38 and regulate the occurrence and development of lung cancer^40^. It can also induce mitochondrial apoptosis in colon cancer cells through a caspase-dependent mechanism^41^. In breast cancer, rutin inhibits P-GP and BCRP pumps non-selectively. It can effectively reverse the resistance of breast cancer cells and restore sensitivity to cyclophosphamide^42^. Chlorogenic acid inhibits tumorigenesis of breast cancer cells by causing mitochondrial dysfunction. It can increase the sensitivity of tumour cells to chemotherapy^43^. Besides, chlorogenic acid can also affect the expression of apoptosis-related genes in A549 human lung cancer cells^44^. In addition to quercetin has been reported to be associated with laryngeal cancer, rutin and chlorogenic acid have been reported to be associated with a variety of cancers. But whether they can treat laryngeal cancer has not been reported, and it may become a potential therapeutic drug.

Our method is different from the common PPI-based network module division method. In the past, network modules were divided based on scale-free topological characteristics to obtain the modules with the best connectivity. In this study, we integrated gene expression data for laryngeal cancer. Modules are divided by co-expressed genes in laryngeal cancer so that they have a better degree of connection in biological functions. After this, we performed a topology analysis on the mapped network and found that important targets are in the center of the network. Therefore, our method not only integrates the patient's disease information but also ensures the high connectivity of the important targets. As the number of patient samples in TCGA, GEO and other public databases increases, more potential targets and active ingredients can be discovered in the future. Besides, our research lacks the validation of animal or cell experiments. In future research, we will perform pharmacodynamic, and toxicity experiments on these candidate components, and in vivo and in vitro experiments such as RT-PCR and western blot will perform biological verification of potential critical genes.

In this study, we analyzed the gene expression data of 109 laryngeal cancer samples and screened to obtain three important targets (MMP1, MMP3, and MMP10). Their expression in tumor samples was significantly higher than in normal samples, and patients in the high expression group had significantly shorter survival times. The components that can be combined with critical targets in HQ are quercetin, rutin, and Chlorogenic acid. These components may be the primary mechanism of the anti-cancer effect of HQ. These findings provide a preliminary research basis for Chinese medicine treatment of laryngeal cancer and offer ideas to related drug design.

## Methods

### Data download and preprocessing

The laryngeal cancer GSE27020 data set was obtained from the GEO database and contained a total of 109 laryngeal cancer samples. The downloaded data needs to be pre-processed before analysis. The clinical information of the samples was extracted based on the Affymetrix Human Genome U133A Array platform, and the probe names were converted into gene names. Finally, a gene expression matrix with row names of gene names and column names of sample names was obtained for subsequent analysis.

### Construction of weighted gene co-expression network

In this study, we use WGCNA package in R software to construct the weighted gene coexpression network of laryngeal cancer. Before constructing network, we need to remove outliers. In this study, the goodsamplesgenes algorithm was used to remove the genes with too many missing values. Then, the hclust algorithm was used to cluster the tissue samples. The top 25% of genes with variance in expression were selected. The remaining samples were used to build a weighted gene co-expression network. The weighting coefficient β is selected according to the criteria of the scale-free network, and the correlation matrix is converted into an adjacency matrix, which is hierarchical clustering based on the value of topology matrix (TOM) value. The dis-TOM is used as the distance measurement of the modules. The minimum module size is set as 10. We use a dynamic hybrid cutting algorithm to identify the module and draw the gene tree^45^.

Then, we calculate the eigenvalue of the module, which is the first principal component obtained by principal component analysis (PCA) of all genes in the module. Screen the modules that are significantly related to clinical characteristics as critical modules. Calculate gene significance (GS) and module membership (MM) to measure the correlation between genes and clinical information^46^.

### Enrichment analysis of critical modules

In order to understand the biological functions of candidate genes, this study maps the genes of critical modules to the online website DAVID (https://david-d.ncifcrf.gov/) for gene ontology (GO) enrichment analysis and KEGG pathway analysis. The items with P < 0.05 were statistically significant.

### PPI network of potential targets of HQ in the treatment of laryngeal cancer

Traditional Chinese Medicine Systems Pharmacology Database and Analysis Platform (TCMSP, https://tcmspw.com/tcmsp.php) includes 499 Chinese herbal medicines registered in the Chinese Pharmacopoeia, 29,384 ingredients, 3,311 targets, and 837 related diseases^47^. From this, all components of HQ and corresponding targets can be obtained. String (version 11.0, https://string-db.org) is a database integrating known and predicted protein–protein interactions, and has the function of visualizing protein–protein interaction network^48^. The PPIs of the HQ target were obtained with medium confidence of 0.4 and then imported into Cytoscape to construct the PPI network of HQ target^49^.

If a node is a potential target of HQ, and it is also in a critical module of a weighted gene co-expression network, this node may be a potential target for HQ treatment of laryngeal cancer^50^. Therefore, we take the HQ PPI network as the background and map the gene modules in the weighted gene co-expression network to the HQ PPI network. The Uniprot database (https://www.uniprot.org/) is used to convert gene names and target names^41^. Therefore, the module based on the weighted gene co-expression network automatically divides the PPI network into multiple modules. The nodes in the critical module are candidate important targets.

### Verification of important targets based on external data sets

GEPIA (https://gepia.cancer-pku.cn/) is an interactive web server for integrated analysis of cancer expression profiling data^52^. It contains 33 malignant tumor RNA sequencing expression data from TCGA and GTEx. In this study, gene expression data from the TCGA and GTEx databases were used as external test sets to verify important targets obtained in the previous screening. Run the "BoxPlots" module to analyze whether important targets have been differentially expressed between laryngeal cancer and normal samples. Kaplan Meier-plotter (https://kmplot.com/analysis/index.php?p=service) is a website for online survival analysis^53^. The survival analysis of important targets was carried out. Under different expression levels, whether the survival time of patients has a significant difference. Enrichment analysis of pathways associated with high or low expression of important targets was performed using GSEA 3.0 software^54^. They are grouped by the expression level of important targets in the GSE27020 dataset. The data set of c2.cp.kegg.v7.0.symbols.gmt in the msigdb database of the GSEA website was selected as the reference gene set. The number of random combinations was set to 1,000.

### Verification of important targets based on molecular docking

Download 3D structures of all molecules in HQ based on the PubChem database^55^. The 3D structure of the target was searched and downloaded from the PDB protein database (https://www.rcsb.org/pdb/home/home.do)^56^. We prefer to select high-resolution crystal structures complexed with corresponding biologically active ligands and use Autodock software for docking^57^. All ligands are removed from the protein receptor complex, plus polar hydrogen atoms and charges. Finally, the compounds in HQ dock with protein crystals in a semi-flexible manner.

## Supplementary information

Supplementary file1 (DOCX 1020 kb)
