# Supplementary material for: Mechanism of Astragalus membranaceus in the treatment of laryngeal cancer based on gene co-expression network and molecular docking
Source: Sci Rep. 2020 Jul 7;10:11184. doi: 10.1038/s41598-020-68093-0 (PMC7340787; doi:10.1038/s41598-020-68093-0)
Supplement: Supplementary file 1 — Supplementary file1 (DOCX 1020 kb) [file 41598_2020_68093_MOESM1_ESM.docx]

Supplementary Data for

**Mechanism of *Astragalus membranaceus* in the treatment of laryngeal cancer based on gene co-expression network and molecular docking**

**Kai Feng Dong^1※^ Meng Qi Huo^4※^ Heng Ya Sun^2^ Tian Ke Li^3^ Dan Li^1*^**

1 The First Hospital of Hebei Medical University, Department of otolaryngology, Shijiazhuang, 050000, China

2 The Third Hospital of Shijiazhuang, Department of otolaryngology, Shijiazhuang, 050011, China

3 The Fourth Hospital of Hebei Medical University, Department of stomatology, Shijiazhuang 050011, China

4 Beijing University of Chinese Medicine, School of Chinese Materia Medica, Beijing, 102488, China

***corresponding author E-mail: 16264688@qq.com**

**^※^The authors contribute equally**


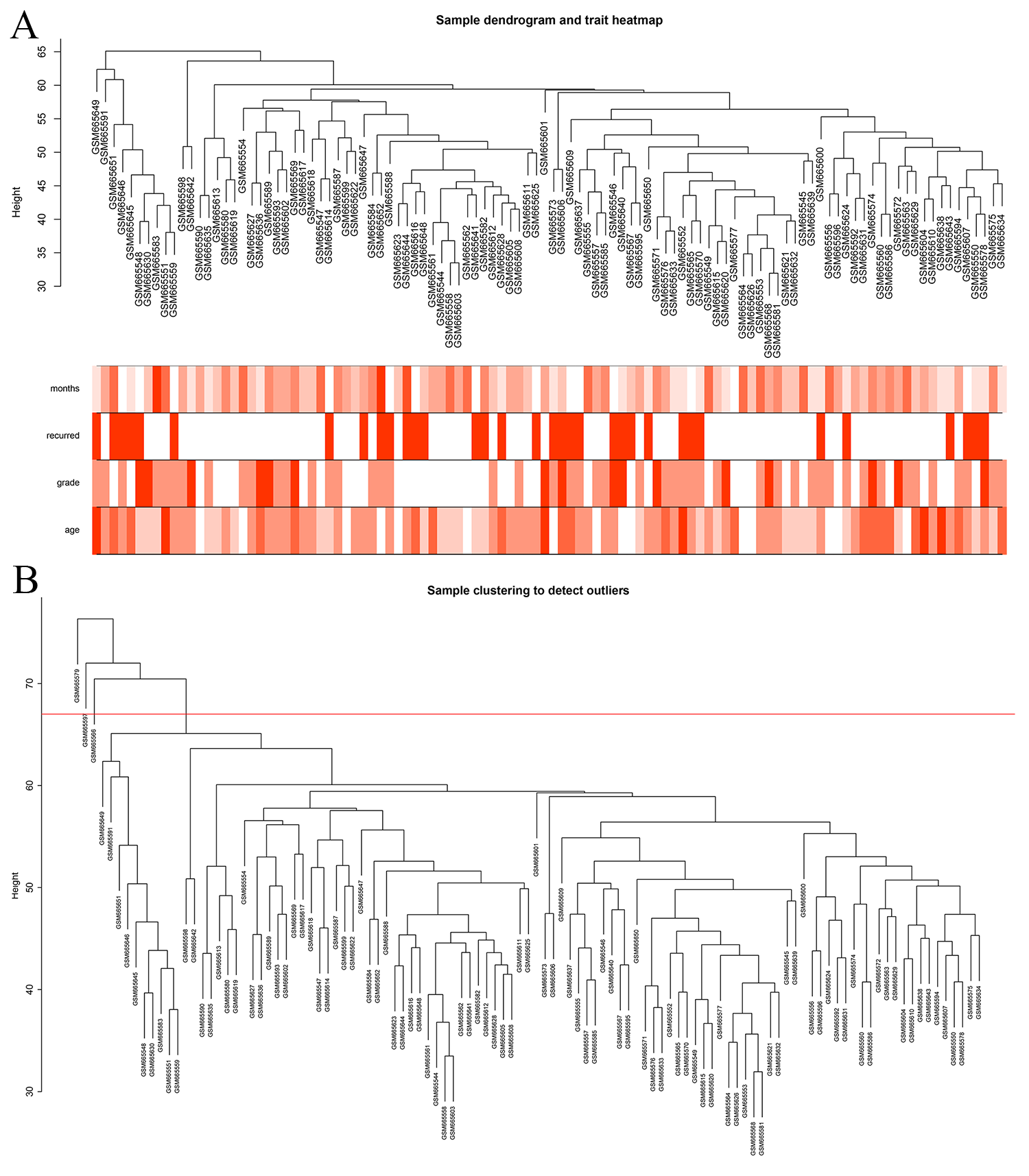


**Figure S1.** Analysis of laryngeal cancer samples based on WGCNA package of R 3.5.1 software (https://www.r-project.org/). (A)The clinical information corresponding to each sample. The darker the color, the larger the value of the item. (B)The clustering was based on the expression data of GSE27020, which contained 109 laryngeal cancer samples. The top 25% of genes with variance in expression were used for construction of weighted gene co-expression network.


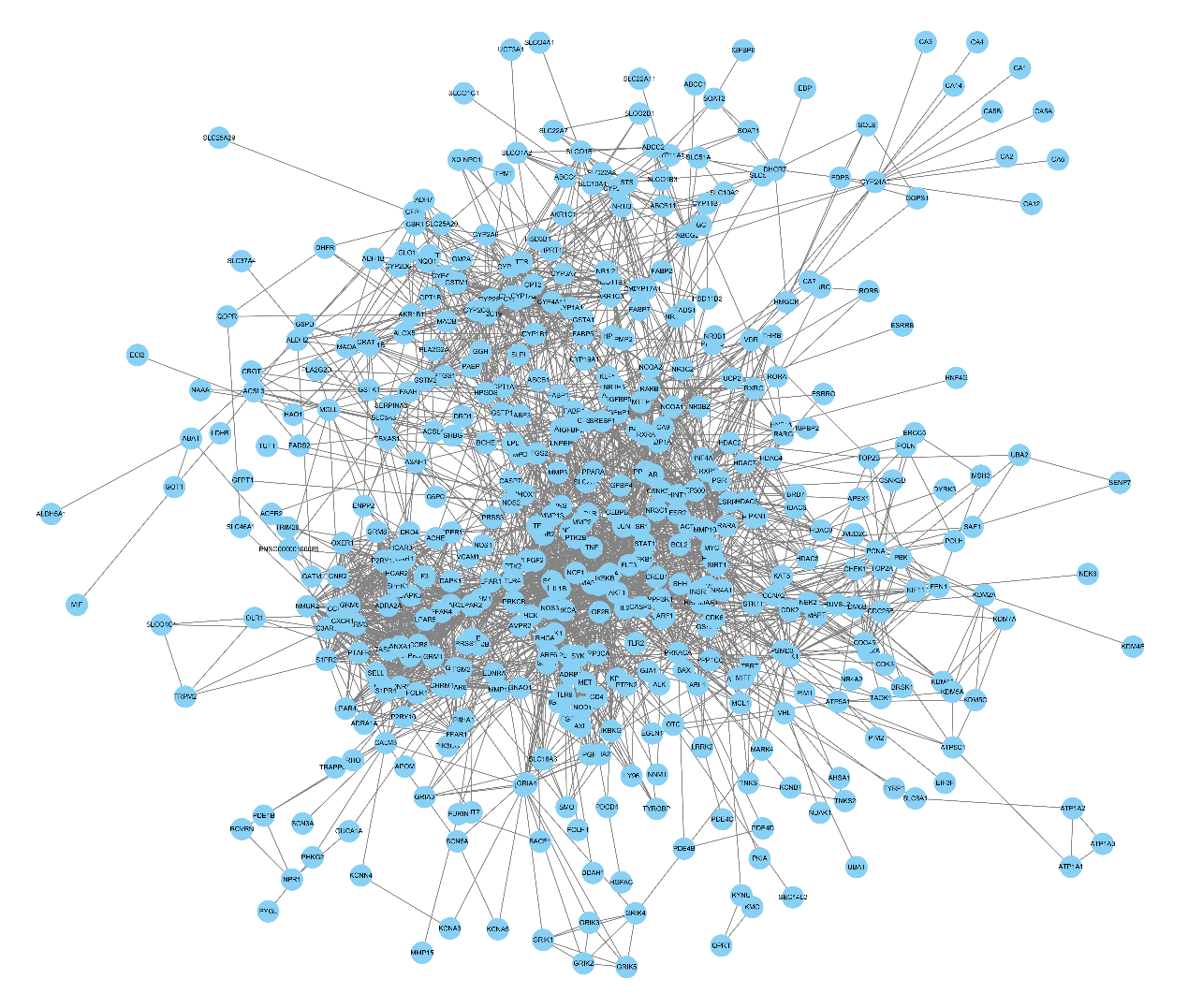


**Figure S2.** Constructing HQ PPI network based on Cytoscape 3.7.0. (https://cytoscape.org/). The blue nodes represent targets in the HQ PPI network, and the black lines represent the PPI between targets.

**Table S1** Enrichment analysis results of the green module

| **Category** | **Term** | **pathway** | **Count** | **%** | **PValue** |
| --- | --- | --- | --- | --- | --- |
| GOTERM_BP_DIRECT | GO:0030198 | extracellular matrix organization | 18 | 15.789474 | 8.74E-15 |
|  | GO:0031581 | hemidesmosome assembly | 8 | 7.0175439 | 3.08E-13 |
|  | GO:0007155 | cell adhesion | 18 | 15.789474 | 6.39E-09 |
|  | GO:0071294 | cellular response to zinc ion | 6 | 5.2631579 | 1.14E-07 |
|  | GO:0045926 | negative regulation of growth | 6 | 5.2631579 | 1.14E-07 |
|  | GO:0022617 | extracellular matrix disassembly | 8 | 7.0175439 | 6.05E-07 |
|  | GO:0007160 | cell-matrix adhesion | 8 | 7.0175439 | 1.92E-06 |
|  | GO:0033627 | cell adhesion mediated by integrin | 5 | 4.3859649 | 2.17E-06 |
|  | GO:0071276 | cellular response to cadmium ion | 5 | 4.3859649 | 3.75E-06 |
|  | GO:0050900 | leukocyte migration | 8 | 7.0175439 | 1.45E-05 |
| GOTERM_CC_DIRECT | GO:0005925 | focal adhesion | 21 | 18.421053 | 1.98E-13 |
|  | GO:0009986 | cell surface | 17 | 14.912281 | 1.59E-07 |
|  | GO:0005615 | extracellular space | 26 | 22.807018 | 3.35E-07 |
|  | GO:0005604 | basement membrane | 8 | 7.0175439 | 4.88E-07 |
|  | GO:0008305 | integrin complex | 6 | 5.2631579 | 5.31E-07 |
|  | GO:0048471 | perinuclear region of cytoplasm | 17 | 14.912281 | 9.74E-07 |
|  | GO:0031012 | extracellular matrix | 12 | 10.526316 | 1.77E-06 |
|  | GO:0005576 | extracellular region | 27 | 23.684211 | 2.60E-06 |
|  | GO:0005578 | proteinaceous extracellular matrix | 11 | 9.6491228 | 5.16E-06 |
|  | GO:0070062 | extracellular exosome | 35 | 30.701754 | 3.24E-05 |
| GOTERM_MF_DIRECT | GO:0005178 | integrin binding | 11 | 9.6491228 | 1.19E-09 |
|  | GO:0031418 | L-ascorbic acid binding | 5 | 4.3859649 | 8.40E-06 |
|  | GO:0005515 | protein binding | 78 | 68.421053 | 1.95E-05 |
|  | GO:0008475 | procollagen-lysine 5-dioxygenase activity | 3 | 2.6315789 | 1.19E-04 |
|  | GO:0043236 | laminin binding | 4 | 3.5087719 | 5.14E-04 |
|  | GO:0098641 | cadherin binding involved in cell-cell adhesion | 9 | 7.8947368 | 5.16E-04 |
|  | GO:0008270 | zinc ion binding | 17 | 14.912281 | 0.0027768 |
|  | GO:0002020 | protease binding | 5 | 4.3859649 | 0.0038894 |
|  | GO:0044325 | ion channel binding | 5 | 4.3859649 | 0.0057896 |
|  | GO:0001948 | glycoprotein binding | 4 | 3.5087719 | 0.0081348 |
| KEGG_PATHWAY | hsa04510 | Focal adhesion | 18 | 15.789474 | 4.48E-12 |
|  | hsa04512 | ECM-receptor interaction | 11 | 9.6491228 | 7.32E-09 |
|  | hsa04151 | PI3K-Akt signaling pathway | 14 | 12.280702 | 1.85E-05 |
|  | hsa04810 | Regulation of actin cytoskeleton | 11 | 9.6491228 | 2.69E-05 |
|  | hsa04978 | Mineral absorption | 6 | 5.2631579 | 5.67E-05 |
|  | hsa05222 | Small cell lung cancer | 7 | 6.1403509 | 1.50E-04 |
|  | hsa05412 | Arrhythmogenic right ventricular cardiomyopathy (ARVC) | 6 | 5.2631579 | 4.26E-04 |
|  | hsa05205 | Proteoglycans in cancer | 9 | 7.8947368 | 6.00E-04 |
|  | hsa05410 | Hypertrophic cardiomyopathy (HCM) | 6 | 5.2631579 | 8.58E-04 |
|  | hsa05414 | Dilated cardiomyopathy | 6 | 5.2631579 | 0.0012003 |

**Table S2** Compound information in HQ

| **ID** | **Name** |
| --- | --- |
| MOL000437 | Hirsutrin |
| MOL000415 | rutin |
| MOL000418 | 3'-Hydroxy-4'-methoxyisoflavone-7-O-beta-D-glucoside |
| MOL000098 | quercetin |
| MOL000442 | 1,7-Dihydroxy-3,9-dimethoxy pterocarpene |
| MOL001955 | Chlorogenic acid |
| MOL000422 | kaempferol |
| MOL000251 | Rhamnocitrin |
| MOL000423 | rhamnocitrin-3-O-glucoside |
| MOL000239 | Jaranol |
| MOL000439 | isomucronulatol-7,2'-di-O-glucosiole |
| MOL000391 | Ononin |
| MOL000379 | 9,10-dimethoxypterocarpan-3-O-β-D-glucoside |
| MOL000436 | (Z)-1-(2,4-dihydroxyphenyl)-3-(4-hydroxyphenyl)prop-2-en-1-one |
| MOL000373 | (2S)-4-methoxy-7-methyl-2-[1-methyl-1-[(2S,3R,4S,5S,6R)-3,4,5-trihydroxy-6-methylol-tetrahydropyran-2-yl]oxy-ethyl]-2,3-dihydrofuro[3,2-g]chromen-5-one |
| MOL000403 | astragalosideII |
| MOL000417 | Calycosin |
| MOL000400 | Flavaxin |
| MOL000390 | daidzein |
| MOL000296 | hederagenin |
| MOL000407 | astragalosideⅣ |
| MOL000409 | AstragalosideIV |
| MOL000433 | (2S)-2-[[4-[(2-Amino-4-oxo-1H-pteridin-6-yl)methylamino]benzoyl]amino]pentanedioic acid |
| MOL000398 | isoflavanone |
| MOL000354 | isorhamnetin |
| MOL000405 | astragalosideⅢ |
| MOL000374 | 5'-hydroxyiso-muronulatol-2',5'-di-O-glucoside |
| MOL000211 | Mairin |
| MOL000377 | 7-hydroxy-3-(2-hydroxy-3,4-dimethoxy-phenyl)chromone |
| MOL000441 | Lupenone |
| MOL000438 | (3R)-3-(2-hydroxy-3,4-dimethoxyphenyl)chroman-7-ol |
| MOL000033 | (3S,8S,9S,10R,13R,14S,17R)-10,13-dimethyl-17-[(2R,5S)-5-propan-2-yloctan-2-yl]-2,3,4,7,8,9,11,12,14,15,16,17-dodecahydro-1H-cyclopenta[a]phenanthren-3-ol |
| MOL000411 | Astraisoflavanin |
| MOL000356 | lupeol |
| MOL000413 | astrachrysoside A |
| MOL000412 | Mucronulatol |
| MOL000393 | Soyasaponin I |
| MOL000392 | formononetin |
| MOL000295 | alexandrin |
| MOL000401 | astragalosideI |
| MOL000376 | 7,2'-dihydroxy-3',4'-dimethoxyisoflavone-7-O-β-D-glucoside |
| MOL000414 | Caffeate |
| MOL000371 | 3,9-di-O-methylnissolin |
| MOL000380 | (6aR,11aR)-9,10-dimethoxy-6a,11a-dihydro-6H-benzofurano[3,2-c]chromen-3-ol |
| MOL000434 | acetylastragaloside I |
| MOL000378 | 7-O-methylisomucronulatol |
| MOL000425 | asernestioside A |
| MOL000054 | Protonated arginine |
| MOL000419 | astrasieversianin XV |
| MOL000427 | asernestioside B |
| MOL000416 | Lariciresinol |
| MOL005928 | isoferulic acid |
| MOL000396 | (+)-Syringaresinol |
| MOL000397 | cis-p-Coumarate |
| MOL000389 | cis-Ferulic acid |
| MOL000431 | coumarin |
| MOL000395 | (2S)-2-Ammonio-4-(carbamimidamidooxy)butanoate |
| MOL000114 | vanillic acid |
| MOL000383 | (2S,3R,4S,5R)-2,3,4,5-Tetrahydroxy-6-oxohexanoic acid |
| MOL000384 | DL-Glucuronic acid |
| MOL000381 | 13-hydroxy-9,11-octadecadienoic acid |
| MOL000424 | alpha-L-Rhamnose |
| MOL000432 | linolenic acid |
| MOL000421 | nicotinic acid |
| MOL000386 | L-(-)-Fucose |
| MOL000429 | (2S)-4-Amino-2-ammonio-4-oxobutanoate |
| MOL000387 | Bifendate |
| MOL000069 | palmitic acid |
| MOL000382 | D-(-)-Arabinose |
| MOL000399 | Docosanoate |
| MOL000061 | Prolinum |
| MOL000131 | Linoleic acid |
| MOL000372 | 3-Hydroxy-2-picoline |
| MOL000420 | DL-Xylose |
| MOL000388 | gamma-aminobutyric acid |
| MOL000394 | choline |
| MOL000430 | betaine |

**Table S3** Docking results of HQ components and MMP1, MMP3, MMP10

| **MMP1** | **Affinity (kcal/mol)** | **MMP3** | **Affinity (kcal/mol)** | **MMP10** | **Affinity (kcal/mol)** |
| --- | --- | --- | --- | --- | --- |
| MOL000437 | -8.6 | MOL000437 | -8.5 | MOL000437 | -8.9 |
| MOL000415 | -9.6 | MOL000415 | -9.3 | MOL000415 | -8.8 |
| MOL000418 | -8.5 | MOL000418 | -9 | MOL000418 | -8.8 |
| MOL000098 | -8.8 | MOL000098 | -9.6 | MOL000098 | -8.7 |
| MOL000442 | -8.8 | MOL000442 | -8.7 | MOL000442 | -8.4 |
| MOL001955 | -9 | MOL001955 | -9 | MOL001955 | -8.3 |
| MOL000422 | -8.3 | MOL000422 | -9 | MOL000422 | -8.3 |
| MOL000251 | -8.1 | MOL000251 | -9 | MOL000251 | -8.3 |
| MOL000423 | -8 | MOL000423 | -8.1 | MOL000423 | -8.3 |
| MOL000239 | -8.1 | MOL000239 | -8 | MOL000239 | -8.3 |
| MOL000439 | -7.5 | MOL000439 | -7.8 | MOL000439 | -8.3 |
| MOL000391 | -8.5 | MOL000391 | -8.8 | MOL000391 | -8.1 |
| MOL000379 | -8.1 | MOL000379 | -7.6 | MOL000379 | -8.1 |
| MOL000436 | -8.7 | MOL000436 | -9.4 | MOL000436 | -8 |
| MOL000373 | -8.8 | MOL000373 | -8 | MOL000373 | -8 |
| MOL000403 | -6.8 | MOL000403 | -7.3 | MOL000403 | -8 |
| MOL000417 | -8.8 | MOL000417 | -9.3 | MOL000417 | -7.9 |
| MOL000400 | -8.2 | MOL000400 | -7.9 | MOL000400 | -7.9 |
| MOL000390 | -8 | MOL000390 | -8.5 | MOL000390 | -7.7 |
| MOL000296 | -8.3 | MOL000296 | -8.3 | MOL000296 | -7.7 |
| MOL000407 | -8.4 | MOL000407 | -8.1 | MOL000407 | -7.7 |
| MOL000409 | -8.5 | MOL000409 | -9.4 | MOL000409 | -7.6 |
| MOL000433 | -8.8 | MOL000433 | -8.7 | MOL000433 | -7.6 |
| MOL000398 | -8.2 | MOL000398 | -7.4 | MOL000398 | -7.6 |
| MOL000354 | -8.7 | MOL000354 | -8.9 | MOL000354 | -7.5 |
| MOL000405 | -7.8 | MOL000405 | -8.5 | MOL000405 | -7.5 |
| MOL000374 | -7.8 | MOL000374 | -8 | MOL000374 | -7.5 |
| MOL000211 | -8.3 | MOL000211 | -6.9 | MOL000211 | -7.5 |
| MOL000377 | -8.1 | MOL000377 | -8.2 | MOL000377 | -7.4 |
| MOL000441 | -8.3 | MOL000441 | -7.2 | MOL000441 | -7.4 |
| MOL000438 | -7.9 | MOL000438 | -9.6 | MOL000438 | -7.3 |
| MOL000033 | -8.7 | MOL000033 | -7.7 | MOL000033 | -7.3 |
| MOL000411 | -8.1 | MOL000411 | -7.4 | MOL000411 | -7.3 |
| MOL000356 | -8.1 | MOL000356 | -7.2 | MOL000356 | -7.3 |
| MOL000413 | -7.5 | MOL000413 | -8 | MOL000413 | -7.2 |
| MOL000412 | -7.8 | MOL000412 | -7.5 | MOL000412 | -7.2 |
| MOL000393 | -6.3 | MOL000393 | -7.1 | MOL000393 | -7.2 |
| MOL000392 | -8.1 | MOL000392 | -8.9 | MOL000392 | -7.1 |
| MOL000295 | -8.1 | MOL000295 | -8.2 | MOL000295 | -7.1 |
| MOL000401 | -6.6 | MOL000401 | -7.3 | MOL000401 | -7.1 |
| MOL000376 | -8 | MOL000376 | -9 | MOL000376 | -7 |
| MOL000414 | -7.1 | MOL000414 | -6.6 | MOL000414 | -7 |
| MOL000371 | -7.6 | MOL000371 | -6.9 | MOL000371 | -6.9 |
| MOL000380 | -7.9 | MOL000380 | -6.7 | MOL000380 | -6.9 |
| MOL000434 | -6.3 | MOL000434 | -6.6 | MOL000434 | -6.9 |
| MOL000378 | -7.7 | MOL000378 | -8.2 | MOL000378 | -6.8 |
| MOL000425 | -7.2 | MOL000425 | -8 | MOL000425 | -6.8 |
| MOL000054 | -6 | MOL000054 | -6.2 | MOL000054 | -6.8 |
| MOL000419 | -8.2 | MOL000419 | -8.3 | MOL000419 | -6.7 |
| MOL000427 | -7.2 | MOL000427 | -7.6 | MOL000427 | -6.7 |
| MOL000416 | -8.7 | MOL000416 | -7.9 | MOL000416 | -6.6 |
| MOL005928 | -7.1 | MOL005928 | -7.4 | MOL005928 | -6.6 |
| MOL000396 | -6.9 | MOL000396 | -7 | MOL000396 | -6.6 |
| MOL000397 | -7 | MOL000397 | -6.9 | MOL000397 | -6.6 |
| MOL000389 | -7.3 | MOL000389 | -7 | MOL000389 | -6.5 |
| MOL000431 | -6.7 | MOL000431 | -6.9 | MOL000431 | -6.5 |
| MOL000395 | -6 | MOL000395 | -6.2 | MOL000395 | -6.5 |
| MOL000114 | -6.6 | MOL000114 | -6.4 | MOL000114 | -6.4 |
| MOL000383 | -5.9 | MOL000383 | -5.8 | MOL000383 | -6.3 |
| MOL000384 | -6.3 | MOL000384 | -5.8 | MOL000384 | -6.1 |
| MOL000381 | -5.9 | MOL000381 | -6.8 | MOL000381 | -5.9 |
| MOL000424 | -5.9 | MOL000424 | -5.5 | MOL000424 | -5.9 |
| MOL000432 | -6.6 | MOL000432 | -6.2 | MOL000432 | -5.7 |
| MOL000421 | -5.9 | MOL000421 | -5.5 | MOL000421 | -5.6 |
| MOL000386 | -5.5 | MOL000386 | -5.2 | MOL000386 | -5.6 |
| MOL000429 | -5.3 | MOL000429 | -5.3 | MOL000429 | -5.5 |
| MOL000387 | -6.1 | MOL000387 | -5.9 | MOL000387 | -5.4 |
| MOL000069 | -5.9 | MOL000069 | -6.1 | MOL000069 | -5.3 |
| MOL000382 | -5 | MOL000382 | -5.1 | MOL000382 | -5.3 |
| MOL000399 | -5.9 | MOL000399 | -6 | MOL000399 | -5.2 |
| MOL000061 | -5.3 | MOL000061 | -5.3 | MOL000061 | -5.2 |
| MOL000131 | -6.5 | MOL000131 | -6.5 | MOL000131 | -5.1 |
| MOL000372 | -4.9 | MOL000372 | -5.5 | MOL000372 | -4.8 |
| MOL000420 | -5.1 | MOL000420 | -4.9 | MOL000420 | -4.7 |
| MOL000388 | -4.3 | MOL000388 | -4.3 | MOL000388 | -4.4 |
| MOL000394 | -3.9 | MOL000394 | -3.7 | MOL000394 | -3.9 |
| MOL000430 | -4.1 | MOL000430 | -3.5 | MOL000430 | -3.8 |
